# Nanotoxicity comparison of four amphiphilic polymeric micelles with similar hydrophilic or hydrophobic structure

**DOI:** 10.1186/1743-8977-10-47

**Published:** 2013-10-03

**Authors:** Bo Zhao, Xue-Qing Wang, Xiao-You Wang, Hua Zhang, Wen-Bing Dai, Jun Wang, Zhen-Lin Zhong, Hou-Nan Wu, Qiang Zhang

**Affiliations:** 1 State Key Laboratory of Natural and Biomimetic Drugs, School of Pharmaceutical Sciences, Peking University, Beijing 100191, China; 2 Medical and Healthy Analytical Center, Peking University, Beijing 100191, China; 3 National Laboratory for Physical Sciences at Microscale and School of Life Sciences, University of Science and Technology of China, Hefei, Anhui 230027, China; 4 Key Laboratory of Biomedical Polymers of Ministry of Education, Department of Chemistry, Wuhan University, Wuhan 430072, China

**Keywords:** Nanotoxicity, Amphiphilic polymeric micelles, J774.A1 cells, Eahy.926 cells, KM mice

## Abstract

**Background:**

Nanocarriers represent an attractive means of drug delivery, but their biosafety must be established before their use in clinical research.

**Objectives:**

Four kinds of amphiphilic polymeric (PEG-PG-PCL, PEEP-PCL, PEG-PCL and PEG-DSPE) micelles with similar hydrophilic or hydrophobic structure were prepared and their *in vitro* and *in vivo* safety were evaluated and compared.

**Methods:**

*In vitro* nanotoxicity evaluations included assessments of cell morphology, cell volume, inflammatory effects, cytotoxicity, apoptosis and membrane fluidity. An umbilical vein cell line (Eahy.926) and a kind of macrophages (J774.A1) were used as cell models considering that intravenous route is dominant for micelle delivery systems. *In vivo* analyses included complete blood count, lymphocyte subset analysis, detection of plasma inflammatory factors and histological observations of major organs after intravenous administration to KM mice.

**Results:**

All the micelles enhanced inflammatory molecules in J774.A1 cells, likely resulting from the increased ROS levels. PEG-PG-PCL and PEEP-PCL micelles were found to increase the J774.A1 cell volume. This likely correlated with the size of PEG-PG-PCL micelles and the polyphosphoester structure in PEEP-PCL. PEG-DSPE micelles inhibited the growth of Eahy.926 cells via inducing apoptosis. This might relate to the structure of DSPE, which is a type of phospholipid and has good affinity with cell membrane. No evidence was found for cell membrane changes after treatment with these micelles for 24 h. In the *in vivo* study, during 8 days of 4 time injection, each of the four nanocarriers altered the hematic phase differently without changes in inflammatory factors or pathological changes in target organs.

**Conclusions:**

These results demonstrate that the micelles investigated exhibit diverse nanotoxicity correlated with their structures, their biosafety is different in different cell model, and there is no *in vitro* and *in vivo* correlation found. We believe that this study will certainly provide more scientific understandings on the nanotoxicity of amphiphilic polymeric micelles.

## Background

Nanomaterials exhibit a wide range of applications in different aspects of human life [1]. In medical and pharmaceutical fields, nanomaterials are packaged into different nanocarriers for biosensing, magnetic resonance imaging, optical detection, and drug delivery systems, among others [2-8]. The accelerating use of nanomaterials increases the likelihood of exposure in humans. Therefore, understanding the biosafety of nanomaterials is a necessity for building nanotechnology systems.

Many *in vitro* and *in vivo* studies have recently been conducted to demonstrate that nanomaterials in direct contact with cell surfaces may lead to several types of damages. Cell visualization appears to be the simplest and the most method of observing direct toxicity on cells. In a study of Yen et al., an increase in the size of the macrophages and a decreasing in cell population were observed after treatment with Au and Ag nanoparticles at ≥10 ppm [9]. Some toxicological *in vitro* studies have reported that nanomaterials can influence reactive oxygen species (ROS) formation [10]. For example, Park et al. reported that the toxicity of ZnO-RT and ZnO-60 was related to ROS formation [11]. Direct cellular toxicity, which may be induced by certain nanomaterials, is another important sign of toxicity. In the study of Tian et al., single-and multi-walled carbon nanotubes (SWCNTs and MWCNTs) were found to be toxic to human cells [12,13]. Certain studies have investigated further influences of nanomaterials on inflammatory factors or protein/gene expression of cells. Yen et al. determined that Au nanoparticles (especially those of a smaller diameter) could up-regulate the expression of the proinflammatory genes interleukin-1 (IL-1), interleukin-6 (IL-6), and tumor necrosis factor (TNF-α) [9].

Compared with *in vitro* toxicity assays, *in vivo* assays are more reflective of the mechanisms of nanomaterial toxicity in the bodies. The common types of *in vivo* nanomaterial toxicity include hematological toxicity, pulmonary toxicity, splenic toxicity, hepatotoxicity and nephrotoxicity [14]. Given the unique qualities of each type of nanomaterial, current research evaluating the toxicity of nanomaterials typically focuses on one aspect of the material properties at a time [14]. The toxicity of most nanomaterials designed for drug delivery systems is correlated with the way they contact with human body. For example, positively charged dendrimers and cationic macromolecules that are mainly restricted to the blood system have been found to interact with blood components, destabilize cell membranes, and induce cell lysis [15-17]. For nanomaterials interacting with human body with other ways, inflammatory changes are a useful means of evaluating toxicity. Poland et al. studied the effect of length on carbon nanotubes (CNT) toxicity via an intraperitoneal injection of MWCNT and observations of carcinogenic mechanisms in the abdominal cavity and the diaphragm [18]. In their study, Poland et al. observed that the longer length (≥20 μm) CNT resulted in an inflammatory response within 24 h, with consequent granuloma formation 7 days after injection. Moreover, additional damage to human bodies induced by the long-term accumulation of nanomaterials has gained increased attention in recent years. For example, Yang et al. studied the toxicity of intravenously injected SWCNTs in the major organs (e.g., liver, lung and spleen) in mice and demonstrated that no histopathological changes were observed in the liver or spleen; the SWCNTs were generally trapped in capillaries and formed aggregates of different sizes in the lung, with some inflammatory cells observed surrounding them [19].

**Figure 1:**
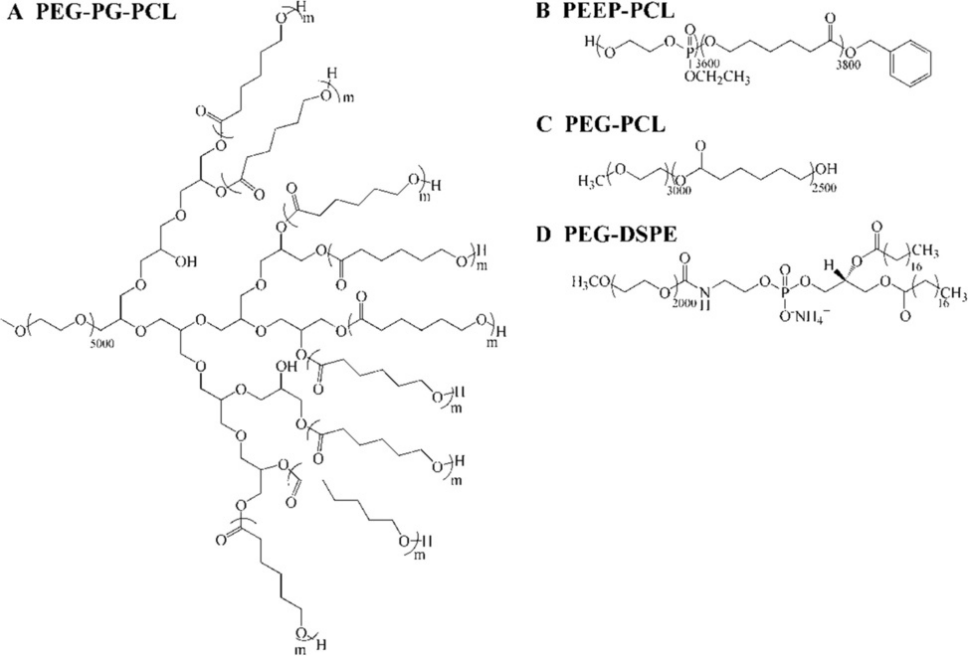
**Chemical constitution of (A) PEG**_**5000**_**-PG**_**300**_**-PCL**_**5700**_**, (B) PEEP**_**3600**_**-PCL**_**3800**_**, (C) PEG**_**3000**_**-PCL**_**2500 **_**and (D) PEG**_**2000**_**-DSPE**_**800**_**.**

Amphiphilic polymers like pegylated polyesters (PEG-PLA, PEG-PLGA, PEG-PCL) are widely used as micelles in drug delivery system. Usually, the inherent physicochemical properties of polymers such as surface charge, hydrophobicity, size, shape, and aggregation tendencies are found to trigger different biological responses [20,21]. Generally, biodegradable polymers with electric neutrality, such as polyesters (PLGA), pegylated polyesters and so on, show low toxicity [22,23]. While, polycations are cytotoxic, inducing hemolysis and complement activation, and polyanions are less cytotoxic but still induce anticoagulant activity and cytokine release [24]. Currently, the main concern on toxicity of polymers is around their metabolism, immunotoxicity and complement activation [20] but there is no systematic safety evaluation has been established for polymers [22]. In this study, we compared the *in vitro* and *in vivo* toxicity of four types of micelles made from poly(ethylene glycol)-polyglycerol-poly(ϵ-caprolactone) (PEG-PG-PCL), poly(ethyl ethylene phosphate)-co-poly(ϵ-caprolactone) (PEEP-PCL), poly(ethylene glycol)-poly(ϵ-caprolactone) (PEG-PCL) and poly(ethyleneglycol)-distearoyl-sn-glycero-phosphoethanolamine (PEG-DSPE) (Figure 1). PEG-PG-PCL, PEEP-PCL, PEG-PCL and PEG-DSPE are all amphiphilic block copolymers. We chose these four types of polymers because they have similar hydrophilic or hydrophobic structure: for example, PEG-DSPE and PEG-PCL have the same hydrophilic segment. When they form into micelles, they have the same shell but a different core; PEEP-PCL and PEG-PCL have the same hydrophobic segment, and their cores are the same, but their shells are different when they form into micelles. Although the structure of PEG-PG-PCL is special, it also has similar shell and core with other three micelles.

PEG-PG-PCL is a novel amphiphilic linear-hyperbranched block copolymer that was successfully synthesized by the Zhong lab [25]. The special functionality of inner porosity and the dense surface of linear-dendritic block copolymers with a hybrid structure could possibly increase the capacity and chemical flexibility of copolymer micelles [26]. When made into micelles, copolymers exhibit more sustained drug release behavior compared with PEG-PCL [25].

PEEP-PCL is another amphiphilic block copolymer recently synthesized by the Wang lab [27]. As polyphosphoesters are degradable and more structurally flexible for physicochemical property adjustments, hydrophilic polyphosphoesters may exhibit interesting properties for drug delivery system design [27]. PEEP-PCL vesicles have been reportedly applied as biodegradable polymer vesicles for drug delivery, revealing that the doxorubicin-loaded vesicles can be successfully internalized by A549 cells to result in enhanced inhibition of A549 cell proliferation [28].

PEG-PCL and PEG-DSPE have been approved by the US Food and Drug Administration (FDA) and have been widely used in drug delivery systems. Li et al. prepared PEG-PCL nanoparticles from different copolymers, and through comprehensive evaluation, they concluded that the tetradrine-loaded nanoparticles exhibited more prominent antitumor effects than free tetradrine [29]. Zeng et al. loaded paclitaxel into PEG-DSPE nanoparticles and observed a higher relative bioavailability compared with the commercial product Taxol, indicating that these PEG-DSPE nanoparticles might serve as a potential sustained release system for poorly water-soluble agents [30].

However, apart from the potential applications of the four micelles prepared from PEG-PG-PCL, PEEP-PCL, PEG-PCL and PEG-DSPE in drug delivery, especially in tumor targeting delivery, the current understanding on the toxicity of these carriers is very limited [31]. A systemic evaluation of these nanomaterials is of great importance for further application in clinical therapeutic areas. Considering that micelles are mostly given via intravenous route, an umbilical vein cell line (Eahy.926) and a kind of macrophages (J774.A1) were used as cell models for *in vitro* toxicity evaluation. While, KM mice were used for *in vivo* toxicity study. Cell morphology and volume detection assays, an inflammatory factor detection assay, a reactive oxygen species (ROS) detection assay, a cell membrane fluidity detection assay, and cytotoxicity and apoptosis assays were conducted in the *in vitro* toxicity study. Complete blood counts, lymphocyte subset detection, detection of plasma inflammatory factors, and histological observations were performed in the *in vivo* toxicity study. Therefore, a comprehensive evaluation system that includes cell toxicity, immune toxicity, hematological toxicity and organs toxicity has been established.

## Results

### Characterization of the micelles

**Figure 2:**
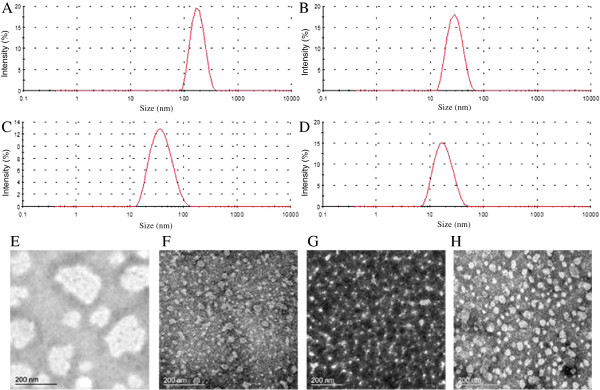
Dynamic light scattering analysis of the particle sizes of PEG-PG-PCL micelles (A), PEEP-PCL micelles (B), PEG-PCL micelles (C), PEG-DSPE micelles (D) and morphological characteristics of PEG-PG-PCL micelles (E), PEEP-PCL micelles (F), PEG-PCL micelles (G), PEG-DSPE micelles (H) observed by TEM.

The sizes and zeta-potential of various micelles measured by Malvern Zetasizer Nano-ZS are shown in Table 1 and the size distribution is shown in Figure 2 (A-D). The particle sizes of the PEEP-PCL, PEG-PCL and PEG-DSPE micelles were 28.0, 43.0 and 17.4 nm, respectively. While micelles prepared from PEG-PG-PCL was 173.4 nm, obviously larger than other three micelles. The polydispersity index (PDI) of the micelles was all below 0.25. The zeta potential of the micelles in PBS was slightly negative. The morphological characteristics of the four types of micelles observed by TEM are shown in Figure 2 (E-H). The micelles were spherical in shape, and their sizes were in accordance to the results of the dynamic light scattering measurements. The CMCs of the four polymers ranged from 0.7 to 2.7 μg/ml, sufficiently low to maintain their micelle states during the experimental process.

**Table 1:** The characteristics of PEG-PG-PCL, PEEP-PCL, PEG-PCL and PEG-DSPE polymeric micelles (mean ± SD, n = 3)

**Preparations**	Particle size (nm)*	PDI*	Zeta potential* (mV)	**CMC (μg/ml)**
PEG-PG-PCL	173.4 ± 5.3	0.044 ± 0.024	-1.27 ± 0.57	0.71
PEEP-PCL	28.0 ± 2.8	0.131 ± 0.102	-4.75 ± 0.74	0.94 [36]
PEG-PCL	43.0 ± 2.8	0.225 ± 0.052	-1.21 ± 0.13	1.01 [36]
PEG-DSPE	17.4 ± 0.8	0.192 ± 0.064	-2.49 ± 0.54	2.69 [36]

*in phosphate-buffered saline (PBS, pH 7.4).

### Cytotoxicity on J774.A1 cells

#### Cell morphology

**Figure 3:**
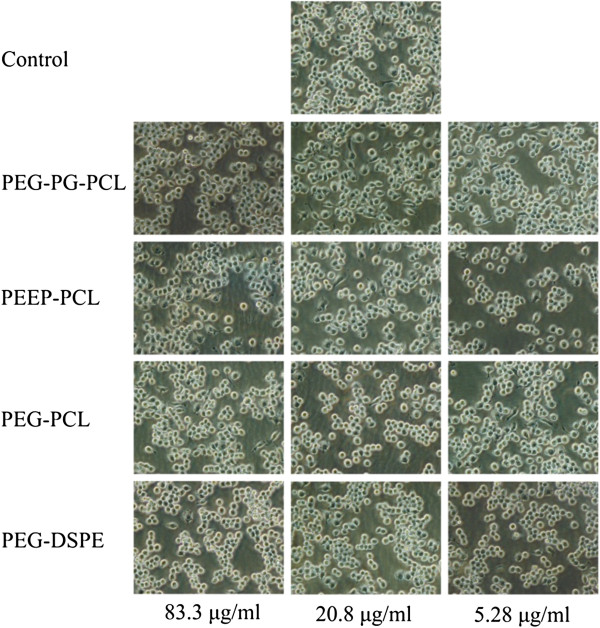
**The morphology of J774.A1 macrophages after treatment with different micelles at different concentrations for 24 h.** The control represents the original morphology of J774.A1 macrophages.

**Figure 4:**
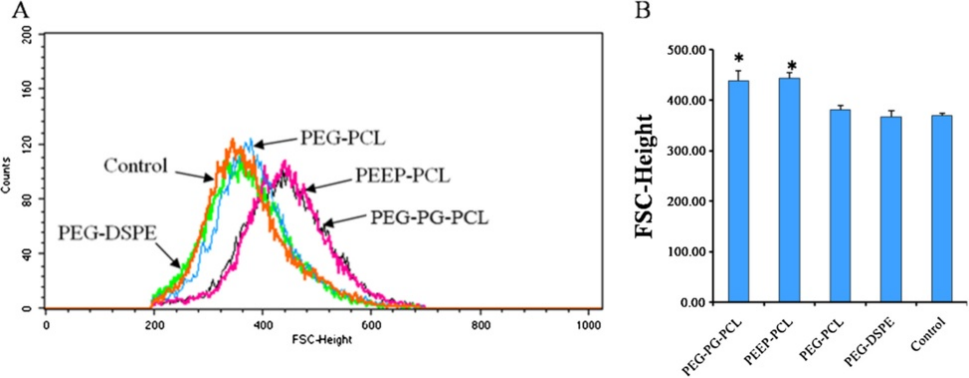
**Flow cytometric measurements of J774.A1 cell volumes treated with different micelles at 83.3 μg/ml for 24 h.** PBS-treated cells served as control. The average cell volume was indicated by the value of FSC-Height. **(A)** Flow cytometric histogram; **(B)** Average value of FSC-Height (n = 3, mean ± SD). *p < 0.01 vs. PBS control.

Figure 3 shows the cell morphology of macrophages treated with various micelles at low (5.28 μg/ml), mid (20.8 μg/ml), and high (83.3 μg/ml) concentrations for 24 h. The cells were all in round shape and appeared healthy. Cells treated with the four kinds of micelles did not exhibit significant differences from the control group. The average size of macrophages treated with various micelles at the concentration of 83.3 μg/ml for 24 h was further detected using flow cytometry. Higher values of FSC-Height represent a larger average cell volume. As Figure 4 demonstrates, after treatment with the micelles, the cells became larger in the PEG-PG-PCL group (439.2 ± 18.6) and PEEP-PCL group (444.2 ± 10.2), whereas the cell sizes in the PEG-DSPE group (367.4 ± 12.2) and PEG-PCL group (380.8 ± 8.1) remained similar to the control group (370.0 ± 4.3).

#### Inflammatory factor level

**Figure 5:**
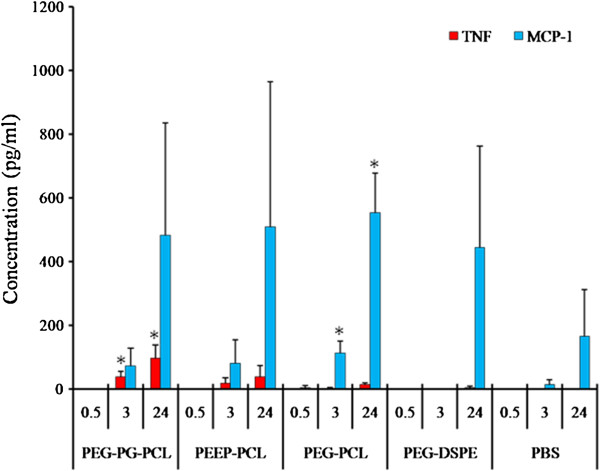
**Inflammatory factor levels in the medium of J774.A1 cells treated with micelles at different time points (0.5, 3 and 24 h).** PBS-treated cells served as controls. The results are given as the mean ± SD, n = 3. *p < 0.05 vs. PBS control.

Four inflammatory factors (IL-6, IL-10, IL-12p70 and IFN-γ) were not detected or were below the limit of detection (20 pg/ml) for all of the four micelles at 0.5, 3 and 24 h. TNF and MCP-1 were not detected at 0.5 h (Figure 5). When the incubation time was increased, TNF and MCP-1 were detected; levels continued to increase with time. All of the micelles led to an obvious growth in MCP-1 compared with the control group treated with PBS at both 3 h and 24 h (excepting PEG-DSPE micelles at 3 h). Similar to their effects on cell size, PEG-PG-PCL and PEEP-PCL induced an increasing level of TNF (119 ± 15 and 46 ± 43 pg/ml), whereas TNF was not detected or was below the detection limit in the other two groups at 24 h.

#### ROS level

**Figure 6:**
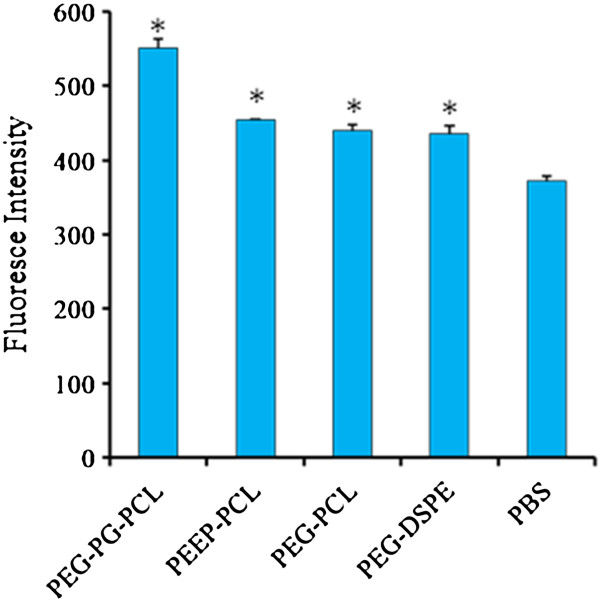
**ROS detection of J774.A1 cells treated with micelles for 24 h.** PBS-treated cells served as controls. The results are given as the mean ± SD, n = 3. *p < 0.01 vs. PBS control.

As shown in Figure 6, the ROS levels of cells treated with various micelles, which were assessed by the fluorescence intensity, were significantly higher than the PBS control. Among the four kinds of micelles, micelles made from PEG-PG-PCL and PEEP-PCL induced the highest and second-highest levels of ROS (550.5 ± 15.6, 453.6 ± 2.1). The results exhibited similarity to the cell size study and the inflammatory factor detection. The fluorescence intensity values of PEG-PCL and PEG-DSPE micelles were 440.4 ± 8.3, 435.9 ± 10.6, respectively, about 18% increase compared with PBS control (372.5 ± 6.5).

### Cytotoxicity study on Eahy.926 cells

#### Cytotoxicity

**Figure 7:**
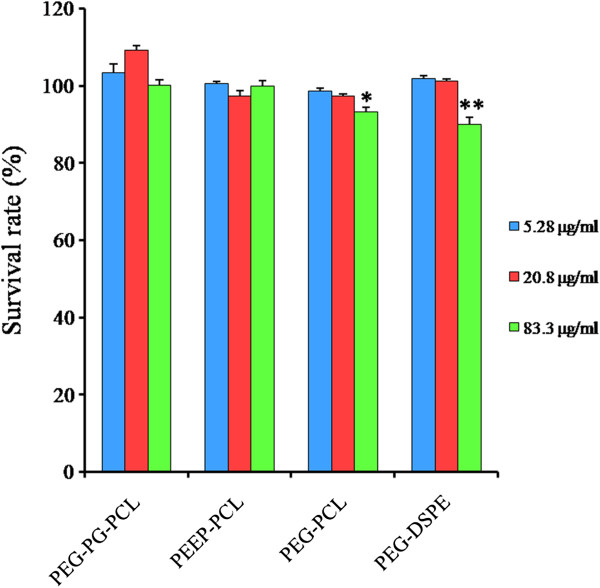
**The survival rates of Eahy.926 cells treated with micelles.** Micelles at three different concentration levels were incubated with cells for 24 h. PBS-treated cells served as controls and the survival rate was considered as 100%. The results are given as the mean ± SD, n = 4. *p < 0.05 vs. PBS control. **p < 0.01 vs. PBS control.

Figure 7 describes the survival rates of cells treated with micelles, which are expressed as the percentage of surviving cells compared with the cells treated with PBS. According to the data, the survival rates of cells treated with various concentrations of micelles were higher than 90%, with the exception of PEG-DSPE (equal to 89.9 ± 1.9%). PEG-PCL in the high-concentration group exhibited the second-lowest survival rate, 93.3 ± 1.2%. Other groups exhibited no significant inhibition of Eahy.926 cells compared with the control group.

#### Apoptosis

**Figure 8:**
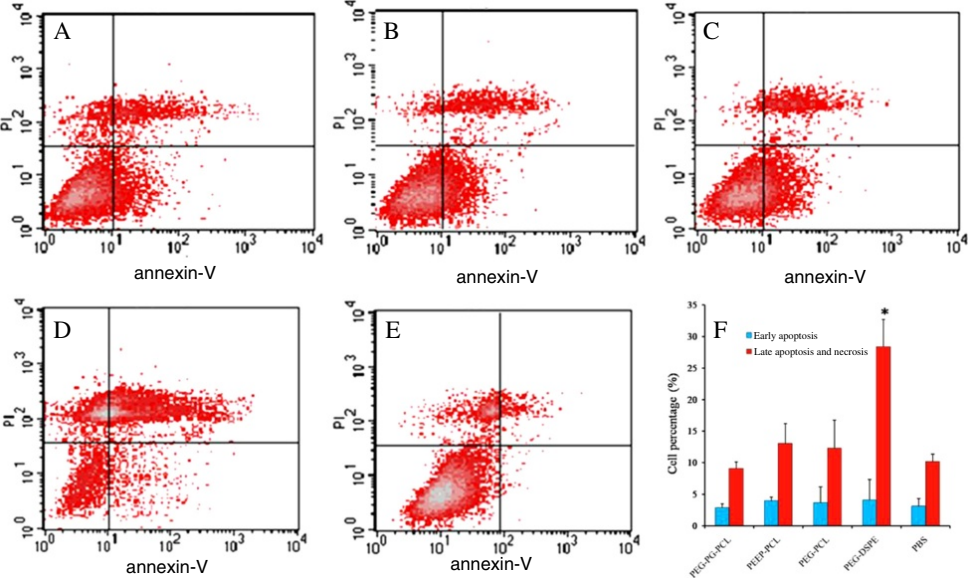
**Apoptosis of Eahy.926 cells induced by PEG-PG-PCL (A), PEEP-PCL (B), PEG-PCL (C), PEG-DSPE (D) and PBS (E) after 24 h. (F)** was the result of apoptotic cell percentage of triplicate tests reported as mean ± SD. *p < 0.01 vs. PBS control.

To confirm the detection of apoptosis, dual staining with Annexin V-FITC and PI method was employed. This method permitted the quantification of early apoptosis, late apoptosis and necrosis. As shown in Figure 8, the number of cells undergoing early apoptosis in each group was below 5% and exhibited no differences from the PBS control. Similar to the cytotoxicity results, the PEG-DSPE group exhibited the highest rate of late apoptotic and necrotic cells (28.3 ± 4.3%), which was significantly different from the PBS control (10.2 ± 1.1%). In the other three groups, no significant differences were observed.

#### Cell membrane fluidity

**Figure 9:**
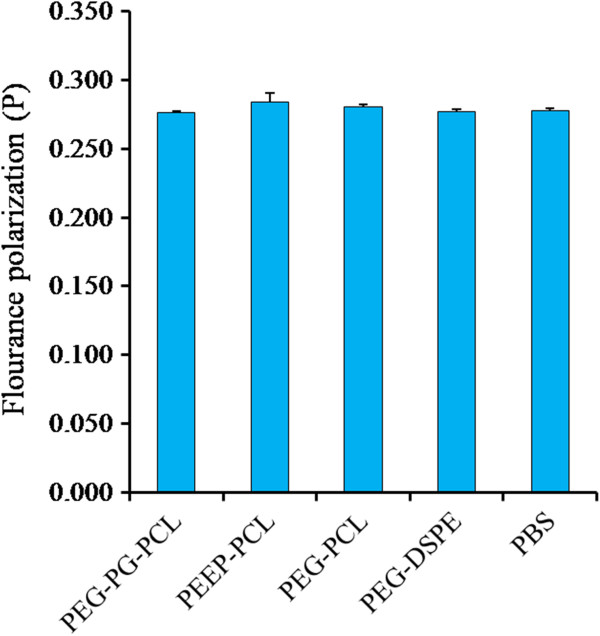
**Membrane fluidity of Eahy.926 cells treated with micelles for 24 h.** PBS-treated cells served as controls. Result are reported as the mean ± SD, n = 3.

To investigate in detail whether the micelles made from the dual-affinity nanomaterials could influence cell membrane fluidity, we analyzed the cell membrane fluidity by DPH assay. DPH is a fluorescent probe commonly used to estimate the bulk of apparent microviscosity of membranes. As the quantum yield of DPH is constant between the various membrane systems, a comparison of apparent membrane microviscosity values can be made using polarization values; a higher polarization value indicates a less fluid cell state. Figure 9 shows the results of this test. Although PEEP-PCL exhibited a higher average P, no significant difference was observed between PEEP-PCL and PBS. For the other groups, the results were similar, indicating that the four kinds of micelles at the concentration 83.3 μg/ml did not influence the cell membrane fluidity of Eahy.926 cells within 24 h.

### Toxicity study on KM mice

#### Complete blood count

**Figure 10:**
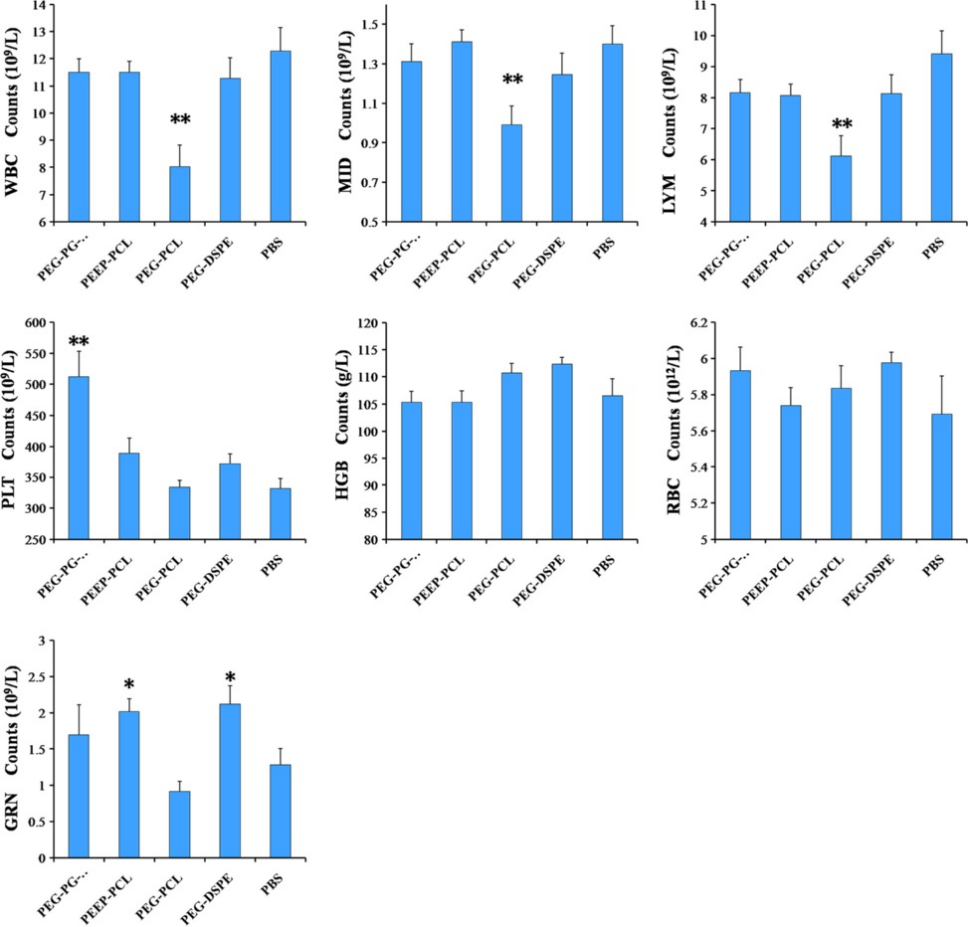
**Complete blood counts of mice after they were injected different micelles.** Saline-treated cells served as controls. The results are reported as the mean ± SE, n = 8. *p < 0.05 vs. saline control, **p < 0.01 vs. saline control.

As shown in Figure 10, none of the micelles induced a significant difference in HGB and RBC counts. The PEG-PCL group, which affected the most factors of the complete blood count, caused three factors to decrease: WBC (8.0 ± 2.5 versus 12.3 ± 2.7, *p* < 0.01), MID (1.0 ± 0.3 versus 1.4 ± 0.3, *p* < 0.01) and LYM (6.1 ± 2.1 versus 9.4 ± 2.4, *p* < 0.01). Compared to the saline (1.3 ± 0.7) control group, PEG-DSPE (2.1 ± 0.8, *p* < 0.01 versus saline) and PEEP-PCL (2.0 ± 0.6, *p* < 0.01 versus saline) both led to a significant increase in GRN counts. Moreover, the PLT counts of the PEG-PG-PCL (512.4 ± 129.3, *p* < 0.01 versus saline) group were significantly higher than the saline (331.6 ± 49.7) control group.

#### Lymphocyte subsets

**Figure 11:**
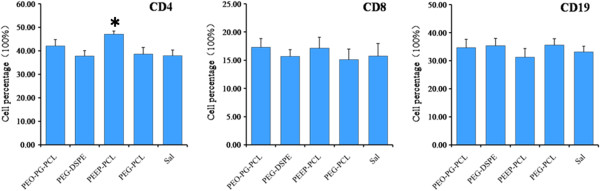
**Lymphocyte subsets of the peripheral blood after the mice were injected with different micelles.** Saline-treated mice served as controls. The results are reported as the mean ± SE for n = 8. *p < 0.01 vs. saline control.

Lymphocyte subsets of the peripheral blood were investigated, and the results are shown in Figure 11. According to the results, CD8^+^ cells and CD19^+^ cell counts exhibited no significant differences in the four micelle groups compared with the saline group. However, the proportion of CD4^+^ cells (47.1 ± 3.9, *p* < 0.01) in the blood of mice treated with PEEP-PCL micelles was higher than that in mice treated with saline (37.9 ± 6.8).

#### Inflammatory factors in plasma

**Figure 12:**
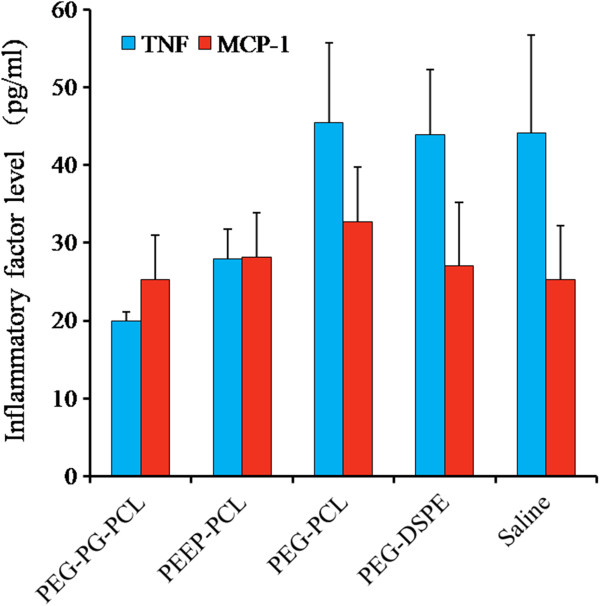
**Inflammatory factors in the plasma after the mice were injected different micelles.** Saline-treated mice served as controls. Result was represented by the mean ± SE, n = 8.

The effect of the micelles on the levels of inflammatory mediators in the plasmic of mice was detected using the CBA technique. Four inflammatory factors (IL-6, IL-10, IL-12p70 and IFN-γ) were not detected or were below the limit of detection (20 pg/ml). TNF and MCP-1 were detected, as shown in Figure 12. No significant differences were observed between any group and the saline group.

#### Histological observations

**Figure 13:**
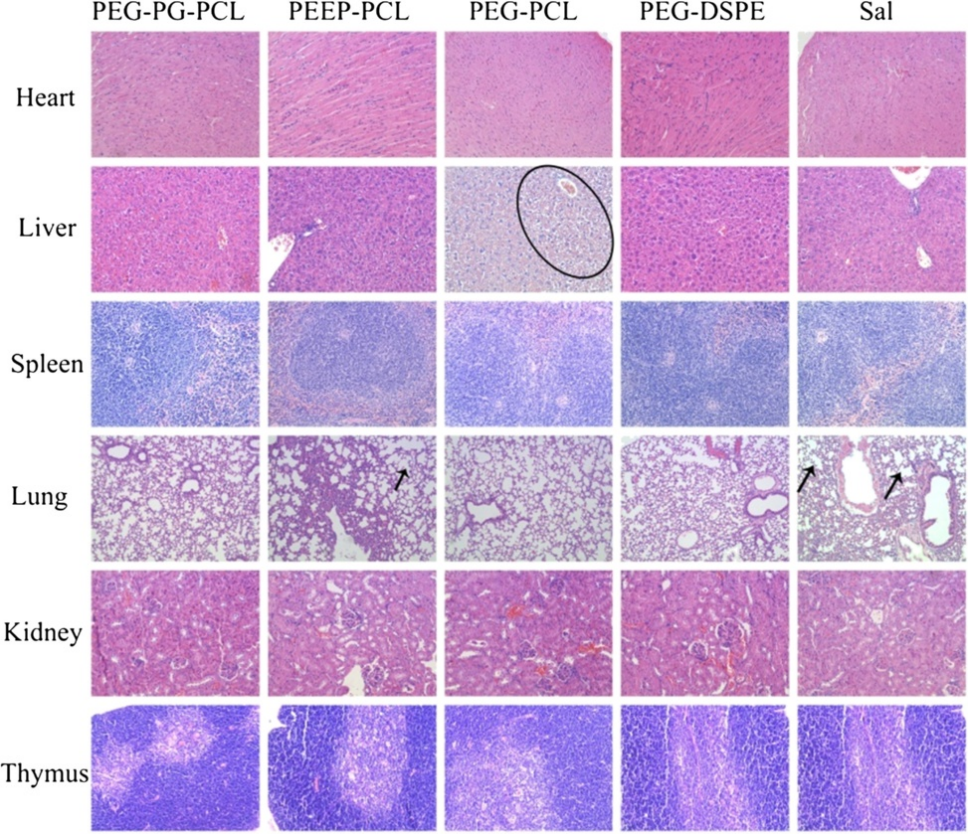
**Histological observations of mice injected with different micelles.** Saline-treated mice served as controls. The lung was observed under 200× magnifications, and other samples were observed under 400× magnifications. Black arrows indicate widened alveolar septum. The black circle indicates widened cytoplasm, lightly stained and highly loose areas in liver.

Histological observations were conducted to determine the organic damage induced by micelles (Figure 13). No clear organic damage was observed in the histological study among all of the exposed groups. However, the alveolar septae were widened, exhibiting blood vessel dilatation and congestion in parts of the lung samples of the PEEP-PCL group and control group; the cytoplasm was widened, lightly stained and highly loose in the liver sample in PEG-PCL group. The organic changes did not represent serious damage and could be a random phenomenon; the control group also exhibited similar changes. The histological observations suggest that these four kinds of micelles cause no significant organic damage but may induce some local changes in certain parts of the organs.

## Discussion

PEG-PG-PCL, PEEP-PCL, PEG-PCL and PEG-DSPE micelles are commonly used for novel nano-preparations. As shown in Figure 1, PEG-PG-PCL has a unique structure of linear-hyperbranched blocks with many arms, whereas PEEP-PCL, PEG-PCL and PEG-DSPE are long chain polymers. Despite the structural differences among these nanomaterials, they all exhibit amphiphilic properties, making it possible for them to form micelles and load drugs. Among these four kinds of micelles, the size of the PEG-PG-PCL micelles was larger than the other three types of micelles; however, the zeta-potentials of these micelles in PBS (pH 7.4) did not differ. The CMCs were all below 3 μg/ml, and the micelle concentrations in our investigation were higher than 5 μg/ml (even *in vivo*, the micelle concentration was approximately 80 μg/ml), indicating the micelles could maintain their micellar state in the present study *in vitro* and *in vivo*.

Two cell lines, J774.A1 and Eahy.926, were used in this study. J774.A1 cells are mononuclear macrophages. The cell line is used as an immune cell model to study the immune responses after stimulation by the micelles. Eahy.926 cells are vascular endothelial cells and are used as a cellular model of the vascular wall. Eahy.926 cells are used to study the toxicity of nanomaterials on the vascular endothelium [32,33]. The reason for the choice of these two cells lies in the application of these nanomaterials in drug delivery. After the micelles are administered intravenously, they (or their original materials and degradation products) typically persist in the blood circulation and have direct access to the blood cells (including immune cells, such as LYM, WBC and macrophages) and vascular endothelial cells.

Macrophages are very sensitive cells in the blood and could respond rapidly to acute nanoparticle toxicity. Normally, macrophages exist in a resting state. When they are stimulated to become active, macrophages grow in volume and are able to engulf foreign antigens and secrete cytokines [34]. As we have demonstrated, J774.A1 cells treated with the micelles exhibited no significant change in shape (Figure 3); however, upon further investigation, we observed an increase in J774.A1 cell size in the PEG-PG-PCL and PEEP-PCL groups (Figure 4). Moreover, PEG-PG-PCL and PEEP-PCL micelles induced TNF, and all four kinds of micelles induced an increase in MCP-1 (Figure 5), indicating that the stimulation process was initiated by the contact between micelles and J774.A1 cells.

During (or after) the stimulation process, protein and ATP content in macrophages increases, oxygen consumption increases significantly, cellular enzymatic activity increases, and the generation of ROS increases [34]. ROS levels significantly increased after treatment with the micelles; PEG-PG-PCL micelles induced the highest level of ROS, and PEEP-PCL micelles induced the second-highest levels (Figure 6). These results suggested that treatment with micelles might elicit an immunological response, resulting from the increasing of ROS levels.

Among all of the micelles, PEG-PG-PCL micelles, followed by PEEP-PCL micelles, most strongly stimulated J774.A1 cells. The reason for difference might lie in the size difference of the micelles and the structure difference of these nanomaterials; phagocytosis generally occurs when particle sizes are larger than 100 nm [35]. The size of the PEG-PG-PCL micelles was 173 nm. Thus, J774.A1 cells may have recognized the PEG-PG-PCL micelles and activated. However, there is no clear reason to explain the phenomenon in PEEP-PCL micelles. We speculated that the interaction might be related to the negatively charge of PEEP-PCL micelles. PEEP, with many phosphoesters, deduced the micelles negatively charged in aqueous solution (-14.4 mV), although it was nearly neutral (-4.75 mV) when added to PBS due to buffer action. As it is reported, negatively charged nanoaprticles can show stronger interaction with cells through nonspecific binding and clustering of the particles on cationic sites on the plasma membrane (that are relatively scarcer than negatively charged domains) compared to nanoparticles with neutral surfaces [23]. In another study on how hydrophilic and hydrophobic structures influence micelle transport in epithelial MDCK cells, PEEP-PCL micelles indeed exhibited unique behavior in terms of endocytosis, exocytosis, organelles colocalization and transcytosis. For example, PEEP-PCL micelles were easier to locate in lysosomes than endoplasmic reticulum in the first ten minutes, while PEG-PCL micelles were concentrated more in endoplasmic reticulum in the first 10 minutes [36].

In the study on Eahy.926 cells, cytotoxicity and apoptosis analyses were conducted. Cytotoxicity and apoptosis analyses are typically used to detect the direct damage of nanocarriers or their degradation products on vascular endothelial cells. The results of the cytotoxicity and apoptosis revealed that among these four kinds of micelles, PEG-DSPE micelles (83.3 μg/ml) significantly inhibited the growth of Eahy.926 cells (Figure 7) and increased the percentage of late apoptotic and necrotic cells (Figure 8). Apoptosis is a form of programmed cell death that occurs through the activation of cell-intrinsic suicide machinery [37]. The increasing percentage of late apoptotic and necrotic cells indicates that PEG-DSPE micelles may trigger apoptosis, leading to the inhibition of cell growth that was observed. We considered that the higher cytotoxicity and apoptosis of PEG-DSPE micelles might result from the higher cellular uptake ability of PEG-DSPE micelles. In our previous study, it was shown that the uptake of the three micelles ranked as PEG-DSPE > PEG-PCL > PEEP-PCL [36]. We considered that as a type of phospholipid with a similar structure to the cell membrane, DSPE had good affinity with cell membrane and deduced higher uptake.

Monitoring cell membrane fluidity is based on the principle that materials exhibit a fat-soluble structure that can insert into the cell membrane and affect its properties. However, in our research, there was no evidence demonstrating that these micelles had any effects on the membrane fluidity of Eahy.926 cells at the given concentration (Figure 9). It is possible that the contact process between the micelles and cells was too long, allowing the cell membrane sufficient time to recover to its origin state, and the interaction between the cell membrane and the micelle process could not be observed.

There were certain differences in the influence of micelles on J774.A1 cells and Eahy.926 cells. J774.A1 cells were stimulated largely by PEG-PG-PCL and PEEP-PCL micelles, but Eahy.926 cells were influenced mainly by PEG-DSPE micelles. We considered the reason for this difference might be associated with the different characteristics of J774.A1 and Eahy.926 cells. As Lewinski et al. reported, from the toxicity study on the effect of C60 exposure under various experimental conditions with different cell lines, the results indeed were related to cell type [38]. J774.A1 cells are of macrophages that can rapidly respond to the environmental changes by secreting various factors. In contrast, Eahy.926 cells are human endothelial cells that exhibit different functions in the human body. As they have unique functions, different reactions to similar stimulations are reasonable.

As we have observed certain physical and chemical changes in the cell models above, it remains to be understood whether these cellular changes occur and cause pathophysiological changes *in vivo*? To answer this question, we conducted a toxicity study in KM mice. There were no obvious body weight and behavior changes post-exposure for all the micelle groups and control (data not shown). Generally, when micelles are injected into vessels of mice, they immediately contact the blood cells and may be delivered to every possible organ and enter cells [33]. We therefore monitored the changes in complete blood cell counts, lymphocyte subset analysis, plasma inflammatory cytokines and changes in target organs, such as the heart, liver, spleen, lung, kidney and thymus. After multiple doses, the micelles of these nanomaterials caused certain changes in blood cells; for example, PEG-PCL micelles decreased the level of WBC, LYM and MID, which are all immune cells that may influence the levels of inflammatory cytokines. PEG-DSPE and PEEP-PCL increased the level of GRN, another type of immune cells, whereas PEG-PG-PCL increased the number of PLT (Figure 10), which may induce hemorrhage, thrombosis or splenomegaly. In the lymphocyte subpopulation analysis, PEEP-PCL induce some increase in the CD4^+^ lymphocyte subpopulation (Figure 11), indicating that PEEP-PCL micelles could stimulate the immune system.

As a result of changes in the circulatory system, other changes in inflammatory factors and organs could follow. However, in our research, rapid changes in inflammatory factors and pathological target organs changes were not observed. One possible explanation might be the time point at which we detected inflammation factors, 24 h after injection, which was long enough for the micelles and inflammation factors to be cleared by the circulation system of mice. Another reason might be that the micelles did stimulate the blood cells and immune cells; however, the stimulation was not strong enough to cause obvious changes in our detection. Alternatively, the micelles of these nanomaterials in circulatory system may have stimulated lymphocytes of blood, which led to cellular stress and subsequent differentiation; this defense system to avoid a further damage on target organs, and the short-term secretion effect of inflammatory cell stress was eliminated after 24 h.

When these *in vitro* (on cells) and *in vivo* (on mice) results were compared comprehensively, we found that the toxicity *in vivo* was not as significant as that *in vitro*. There are several possible reasons: (1) The micelles *in vivo* exist mainly in the blood system, which is a dynamic environment, whereas the *in vitro* studies are performed in a relatively static environment, thereby providing more chances for micelles to contact cells. (2) The body’s innate ability to self-regulate is much more prevalent than regulation in cultured cells.

## Conclusions

In this study we prepared PEG-PG-PCL, PEEP-PCL, PEG-PCL and PEG-DSPE micelles and compared their nanotoxicity on J774.A1 cells, Eahy.926 cells and mice. It was indicated that all micelle systems induced a change in inflammatory factors, potentially as a result of the increased level of ROS. PEG-PG-PCL micelles and PEEP-PCL micelles led to an increase in cell volume. This phenomenon likely correlated with the size of PEG-PG-PCL micelles and the polyphosphoester structure in PEEP-PCL. Besides, PEG-DSPE micelles inhibited the growth of Eahy.926 cells by inducing apoptosis. No evidence was found for cell membrane changes after treatment with these micelles. Likely due to the direct injection into veins, these nanocarriers were found to influence blood components differently. However, these changes in the blood did not induce significant alterations in inflammatory factors and pathology of major mouse organs. The difference between the *in vitro* and *in vivo* results indicates that the *in vitro* toxicity may not occur *in vivo*, probably because the animal body can protect against certain toxicities. Additionally, there may be other toxicity-related reactions found *in vivo* that were not observed *in vitro* due to the unknown reasons. Because there is currently no standard for nanotoxicity, it is difficult for us to conclude whether the observed changes are serious or negligible. In general, it is demonstrated that the micelle systems tested here show diverse nanotoxicity correlated with their structures and their biosafety is different in different cell model. This study will certainly provide more scientific understandings on the nanotoxicity of amphiphilic polymeric micelles.

## Materials and methods

### Materials

Poly(ethylene glycol)-polyglycerol-poly(ϵ-caprolactone) (PEG_5000_-PG_300_-PCL_5700_, Mw 11000) was synthesized by the Department of Chemistry, Wuhan University (Wuhan, Hubei, China). Poly(ethyl ethylene phosphate)-co-poly(ϵ-caprolactone) (PEEP_3600_-PCL_3800_, Mw 7400) was synthesized by the National Laboratory for Physical Sciences at the Microscale and School of Life Sciences, University of Science and Technology of China (Hefei, Anhui, China). Poly (ethylene glycol)-poly (ϵ-caprolactone) (PEG_3000_-PCL_2500_, Mw 5500) was purchased from Advanced Polymer Materials, Inc. (Montreal, QC, Canada). Poly(ethyleneglycol)-distearoyl-sn-glycero-phosphoethanolamine (PEG_2000_-DSPE_800_, Mw 2800) was obtained from the NOF Corporation (Japan). Cytometric bead array (CBA) mouse inflammation kit was purchased from BD Biosciences (San Jose, CA, US). Cell counting kit-8 (CCK-8) was supplied by Dojindo (Tabaru, Mashikimachi, kamimashiki gun Kumamoto, Japan). 1,6-diphenyl-1,3,5-hexatriene (DPH) and 2′,7′-dichlorofluorescin diacetate (DCFH-DA) was purchased from Sigma (St. Louis, MO). Annexin V-fluorescein-isothiocyanate (Annexin V-FITC) and propidium iodide (PI) was purchased from Biomiga (Santiago, US). Antibodies CD19-FITC, CD4-FITC, CD8a-phycoerythrin (CD8a-PE) and CD3e-peridinin chlorophyll protein (CD3e-PerCP) were purchased from BD Biosciences (San Jose, CA, US).

### Cells

J774.A1 murine macrophages (from the Institute of Basic Medical Science, China Academic Medical Science, Beijing, China) and Eahy.926 human umbilical vein cell line (from the Type Culture Collection of the Chinese Academy of Sciences, Shanghai, China) were cultured in high-glucose Dulbecco’s modified Eagle’s medium (DMEM; Gibco, Carlsbad, CA, USA) containing 10% fetal calf serum, 1% penicillin- streptomycinamphotericin B solution. The cells were cultured in an incubator at 37°C in an atmosphere of 5% CO_2_/95% air and 99% relative humidity and passaged twice a week.

### Animals

Male KM mice (18-22 g) were obtained from Peking University Animal Center, Beijing, China. They were housed in plastic cages (4 mice/cage) and kept on a 12 h light/dark cycle. Food and water were provided ad libitum. All animal experiments were performed in compliance with the institutional ethics committee regulations and guidelines on animal welfare (Animal Care and Use Program Guidelines of Peking University).

### Preparation and characterization of micelles

#### Preparation of PEG-PG-PCL and PEEP-PCL micelles

PEG-PG-PCL and PEEP-PCL micelles were prepared using a solvent dispersing method. Briefly, 4 ml deionized water was slowly dropped into 1 ml stirring acetonitrile containing 5 mg PEG-PG-PCL or 5 mg PEEP-PCL. Afterward, the mixture was evaporated to approximately 3 ml in a 37°C water bath. Next, the same volume of 2× phosphate-buffered saline (PBS) was added to the mixture, and a PEG-PG-PCL or PEEP-PCL micelle solution with PBS (pH 7.4) was obtained.

#### Preparation of PEG-DSPE and PEG-PCL micelles

To prepare PEG-DSPE and PEG-PCL micelles, the thin-film dispersion method was used [39]. Briefly, 5 mg PEG-DSPE or PEG-PCL was dissolved in 1 ml acetonitrile, and subsequently evaporated under a vacuum until a thin lipid film formed. The lipid film was hydrated with 1 ml PBS (pH 7.4) at 25°C for PEG-DSPE micelles and at 60°C under a sonicator for PEG-PCL micelles. Lastly, 5 ml additional PBS (pH 7.4) was added to the mixture, and PEG-DSPE or PEG-PCL micelles were prepared.

#### Characterization of micelles

The particle size and Zeta potential were determined by dynamic light scattering (Malvern Zetasizer Nano-ZS, Malven Instruments, Malven, UK). The dispersion medium used in characterization of the micelles was PBS (pH 7.4). The intensity was used for calculating the size of the micelles. The morphology of the micelles was characterized by transmission electronic microscopy (TEM, JEOL, JEM-200CX, Japan). The critical micelle concentration (CMC) of different polymers was determined by pyrene fluorescence probe spectrometry [36].

### Toxicity study on J774.A1 cells

#### Cell culture

J774.A1 cells were seeded in 6-well culture plates at an initial density of 2 × 10^5^ cells per well. After 24 h of culture, the medium was changed to fresh medium containing different micelles at the reported concentrations, and the cells were cultured for another predetermined time. Cells treated with PBS were used as controls.

#### Cell morphology and volume detection

For cell morphology visualization, the cells were treated with micelles at different concentrations (5.28, 20.8 and 83.3 μg/ml) for 24 h and were observed at 200 × magnifications with a Provis microscope (Olympus) [9].

For cell volume detection, the cells were treated with micelles at 83.3 μg/ml for 24 h. Next, the cells were rinsed three times to remove residual micelles in the medium before the cells were detached by trypsin. Then, the cells were centrifuged and resuspended in 400 μl PBS. The number and average size of the cells were determined by a flow cytometer (Beckman Coulter Reagents, USA).

#### Detection of inflammatory factors

To detect inflammatory factors, we used a technique known as cytometric bead array (CBA) (San Jose, CA, US) [40]. Capture beads bound to antibodies of six different fluorescence intensities were used to detect six inflammatory factors (interleukin-6 (IL-6), interleukin-10 (IL-10), interferon-γ (IFN-γ), tumor necrosis factor (TNF), interleukin-12p70 (IL-12p70), and monocyte chemotactic protein 1 (MCP-1)). When the capture beads and detector reagent are incubated with samples, sandwich complexes are formed. These complexes can be measured using flow cytometry to identify particles with the fluorescence characteristics of both the bead and the detector. In detail, the cells were treated with different micelles at a final concentration of 83.3 μg/ml, and 50 μl of the medium in each sample cells was collected at 0.5, 3 and 24 h and frozen at -20°C before use. Before detection, the mouse inflammation standards were prepared from 0 to 5000 pg/ml. Subsequently, six kinds of beads were mixed and vortexed thoroughly. Fifty microliters of the mouse inflammation standard dilutions or 50 μl of samples was added to 50 μl of the mixed capture beads. Fifty microliters of the mouse inflammation PE detection reagent was added to each of the mixtures above. After incubating the assay tubes for 2 h at room temperature (protected from light), 1 ml wash buffer was added to each tube and centrifuged at 200 g for 5 min. Lastly, the supernatant was carefully aspirated and discarded from each assay tube, and an additional 300 μl of wash buffer was added to each assay tube to resuspend the bead pellet before data acquisition with flow cytometry. The data are expressed as average protein concentrations in each group and presented as mean ± SD (n = 3).

#### Detection of ROS

The cells were treated with different micelles at a final concentration of 83.3 μg/ml for 24 h. After the cells were trypsinized, centrifuged, and resuspended, different groups were preincubated for 30 min with 1 × 10^-7^ mol/L of DCFH-DA in an incubator at 37°C with horizontal agitation. DCFH-DA diffused into cells and was hydrolyzed into nonfluorescent 2′-7′-dichlorofluorescin (DCF). DCF fluorescence was detected at 530 nm after excitation of cells at 488 nm using flow cytometry (Beckman Coulter Reagents, USA) [41]. The results are expressed as average fluorescence intensity of cells in each group (n = 3).

### Toxicity study on Eahy.926 cells

#### Cytotoxicity

Cytotoxicity was measured using the CCK-8 assay, which is based on the conversion of water-soluble tetrazolium salt, WST-8 (2-(2-methoxy-4-nitrophenyl)-3-(4-nitrophenyl)-5-(2,4-disulfophenyl)-2H-tetrazolium, monosodium salt), to a water-soluble formazan dye upon reduction in the presence of an electron carrier by dehydrogenases. Briefly, the cells were treated with various concentrations (5.28, 20.8 and 83.3 μg/ml) of PEG-PG-PCL, PEEP-PCL, PEG-PCL and PEG-DSPE micelles for 24 h at 37°C or treated with medium as control. Subsequently, 10 μl of WST-8 solution was added to the medium and incubated for an additional 2 h at 37°C. The absorbance was determined using a Thermo Scientific multiscan FC microplate photometer at the wavelength of 450 nm. The data are expressed as the percentages of surviving cells compared to the survival of the control group (cells treated with medium as 100%) and presented as mean ± SD (n = 4).

#### Apoptosis

Apoptosis analyses were performed by the Annexin V-FITC and PI double staining method [42]. Double staining for both Annexin V-FITC binding and cellular DNA using PI was performed as follows: Cells (5 × 10^6^ cells per ml) treated with different micelles at 83.3 μg/ml for 24 h were centrifuged, and the resulting pellet was resuspended in PBS. Cells in PBS were centrifuged again and the pellet was resuspended in binding buffer (10 mM 2-[4-(2-Hydroxyethyl)-1-piperazinyl]ethanesulfonic acid (HEPES)/NaOH, pH 7.4, 150 mM KCl, 1 mM MgCl_2_ and 1.8 mM CaCl_2_). Annexin V-FITC was added to the pellet, resulting in a final concentration of 2.5 μg/ml. The mixture was incubated in the dark at room temperature for 10 min. PI (1 mg/ml) was added 5 min before flow cytometric analysis, resulting in a final concentration of 50 μg/ml. The data are expressed as the percentages of early apoptotic, late apoptotic and necrotic cells and presented as mean ± SD (n = 3).

#### Cell membrane fluidity

DPH was used to monitor the plasma membrane fluidity of Eahy.926 cells [43]. Labeling of treated cells by the method above was performed by incubating 2 × 10^-6^ mol/L DPH in 2,5-dimethylfuran. The suspension was allowed to equilibrate for 30 min at 37°C. Fluorescence polarization was measured using the Perkin Elmer 650-40 spectrofluorometer. The degree of fluorescence polarization, P, defined in the following equation, was directly recorded.

P = (I_v_-I_h_)/(I_v_ + I_h_), where I_v_ and I_h_ are the emission intensities passing polarizers oriented vertically and horizontally, respectively, with respect to the vertical polarization vector of the exciting light. The data were represented by the average P value of each group and presented as mean ± SD (n = 3).

### Toxicity study on KM mice

#### Micelles administration and sampling

Forty mice were randomly divided into five groups (8 mice/group). Four kinds of micelles (i.e., PEG-PG-PCL, PEEP-PCL, PEG-PCL and PEG-DSPE micelles) and saline (control group) were i.v. (tail vein) injected every 48 hours, 4 times (8 mg/kg each time), respectively. Body weight and behaviors were recorded every other day post-exposure. On the 8th day post-exposure, the mice were sacrificed, and blood/organ samples were collected. The blood samples were used to complete blood counts and lymphocyte subset analyses. The organs (liver, lung, heart, kidney, thymus and spleen) were obtained for pathological observation.

#### Complete blood count

Blood samples were collected by a certified phlebotomist from orbit into blood collection tubes [44]. A mixture of 20 μl whole blood and 1 ml dilute solution was used to assess the following: white blood cells (WBC), intermediate cells (MID), lymphocytes (LYM), platelets (PLT), hemoglobin (HGB), red blood cells (RBC) and granulocytes (GRN). All blood samples were tested using a MEK-6318 K (Nihon Kohden, Japan) autoanalyzer.

#### Lymphocyte subsets

The peripheral blood (1 ml/mouse) was collected in heparinized tubes for all of the experiments [45]. One hundred microliters of blood was incubated in the dark for 30 min with the following antigens: 5 μl CD3e-PerCP, 2 μl CD4-FITC and 5 μl CD8a-PE. An additional 100 μl was incubated with 5 μl CD3e-PerCP and 2 μl CD19-FITC. Using the double labeling technique, CD3^+^CD4^+^, CD3^+^CD8^+^, and CD3^+^CD19^+^ cell subsets were assayed. The erythrocytes were lysed with lysing solution (HLA-B27 Kit, BD), and the cells were washed twice with PBS. The samples were counted using a flow cytometer (Beckman Coulter Reagents, USA).

#### Detection of inflammatory factors in plasma

Plasma samples were collected from centrifuged peripheral blood, 100 μl per sample. The same method of cytometric bead array was performed to detect the inflammatory factors as above described.

#### Histological observations

For histological observations, organ samples (heart, liver, spleen, lung, kidney and thymus) were fixed in a 4% formaldehyde solution, paraffin-embedded, thin-sectioned, and mounted on glass microscope slides using the standard histopathological techniques. The mounted sections were stained with hematoxylin-eosin (H&E) and examined using light microscopy.

#### Statistical analysis

All data are presented as the means of individual observations with the standard deviation. The significance has been calculated using one-way ANOVA. The differences were considered to be significant if *p* < 0.05.

## Competing interests

The authors declare that they have no competing interests.

## Authors’ contributions

BZ and XYW performed research. BZ, XQW, HNW, QZ designed & analyzed & interpreted data, and wrote the manuscript. HZ and WBD interpreted data and reviewed the manuscript. JW and ZLZ provided PEEP-PCL and PEG-PG-PCL polymers. All authors read and approved the final manuscript.
